# 1765. Distribution of Rickettsia species among hospitalized cases and ticks in Pavlodar region, Kazakhstan, 2019

**DOI:** 10.1093/ofid/ofad500.1596

**Published:** 2023-11-27

**Authors:** Yekaterina V Bumburidi, Dmitriy V Berezovskiy, Bakhytkul Zhakipbayeva, Roberta Horth, Yekaterina Ostapchuk, Galina Zemtsova, Zhanna Berdygulova, Willam Nicholson, Zhanna Shapiyeva

**Affiliations:** CDC/Central Asia office, Almaty, Almaty, Kazakhstan; U.S. Centers for Disease Control and Prevention, Central Asia Office, Almaty, Kazakhstan, Almaty, Almaty, Kazakhstan; U.S. Centers for Disease Control and Prevention, Central Asia Regional Office, Almaty, Kazakhstan, Almaty, Almaty, Kazakhstan; US Centers for Disease Control and Prevention, Dulles, Virginia; Almaty Branch of the National Center for Biotechnology, Almaty, Almaty, Kazakhstan; CDC, Atlanta, Georgia; Almaty Branch of the National Center for Biotechnology, Almaty, Almaty, Kazakhstan; CDC, Atlanta, Georgia; SPC SEEM - the branch of NCPH, Almaty, Almaty, Kazakhstan

## Abstract

**Background:**

Spotted Fever Group Rickettsioses (SFGR) is a group of neglected, life-threatening zoonotic diseases caused by closely related bacteria spread primarily by ticks. Treatment is available if identified, but testing for *Rickettsiae* is limited. Little is known about SFGR prevalence and distribution of *Rickettsiae* species in Kazakhstan. This knowledge can help inform SFGR prevention and treatment efforts.

**Methods:**

We conducted sentinel surveillance from April to October 2019 in six hospitals in the Pavlodar region, an area where several SFGR cases had previously been detected. We interviewed and collected samples from all consenting patients ≥ 6 years old with SFRG symptoms. Parents consented for patients < 18 years old. Samples included skin lesions and two serum samples at the acute stage (AS) and convalescent stage (CS). We classified participants as having acute SFGR if they had either: (1) positive PCR, or (2) a four-fold increase in IFA IgG titers from AS to CS samples, or (3) IgG titers (≥ 1:64) in an AS sample. We also collected ticks using dragging or flagging of vegetation and tested them using real-time PCR.

**Results:**

Of 105 people enrolled, 69% had acute SFGR (fig. 1). Incidence rates per 100,000 population at sentinel sites ranged from 7 to 50. Common signs were fever (100%), headache (89%), rash (75%), and myalgia (58%). *R. sibirica* (40%), *R. raoultii* (39%), and *R. slovaca* (7%) were identified in participants (tab. 1). Participants with *R. sibirica* were more likely than participants with *R. raoultii* to have eschar (80% vs. 7%, p < 0.01, respectively) and rash (90% vs. 61%, p=0.01, respectively). Doxycycline was administered to 64% of participants, and nobody died.

*R. raoultii* and *R. spp* were identified in 9% of *Dermacentor reticulatus* ticks (n=610) and 7% of *Dermacentor marginatus* ticks (n=349). *R. sibirica* was identified in 1% of *D. marginatus* ticks. *S. slovaca* was not detected in ticks (fig. 2).

**Figure 1.**
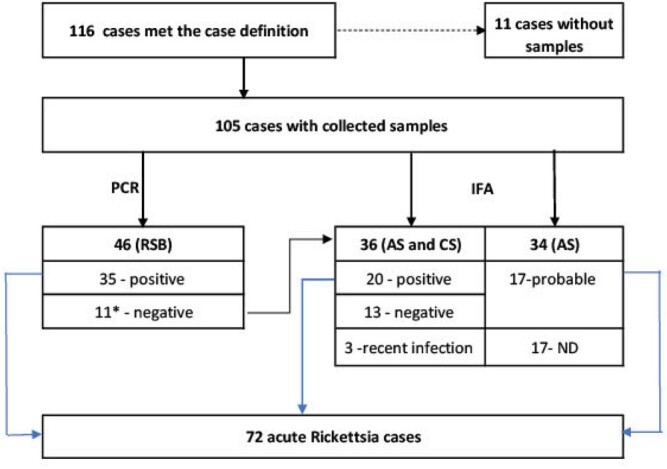
Patient enrollment, specimen collection and testing results, Pavlodar region, 2019. PCR: polymerase chain reaction; RSB: rash skin biopsy or /and eschar or/and swab from eschar samples; IFA: immunofluorescence assay; AS: acute serum collected at acute phase; CS: convalescent serum collected at convalescent phase; ND: not defined. *11 – PCR negative tested with IFA. Of which, 5 had both AS and CS (results: 2-positive, 2 -negative, 1-recent infection) and 6 had AS only (results: 2-probable, 4 not defined).

**Table 1.**
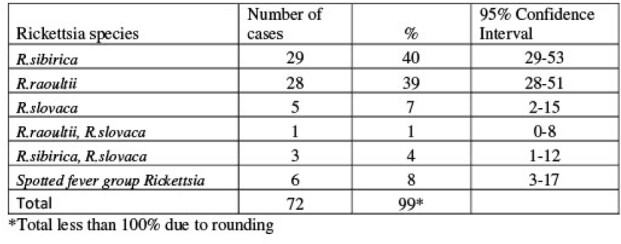
Rickettsia species identified from hospitalized cases (n=72)

**Figure 2.**
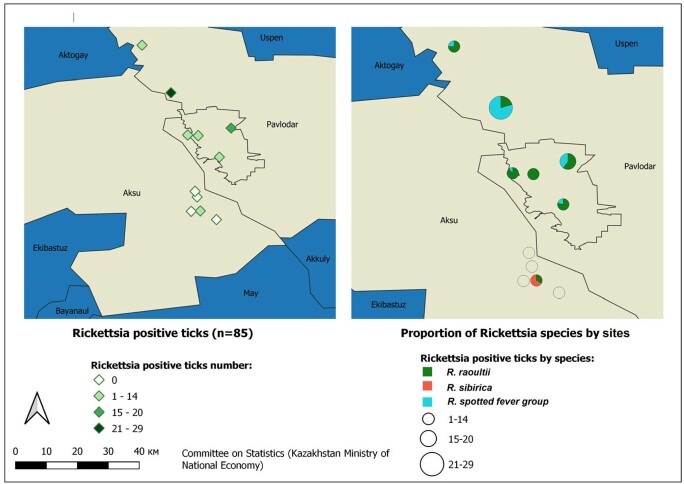
Rickettsia species in ticks by selected sites (Aksu and Pavlodar districts), Pavlodar region, 2019

**Conclusion:**

There was a two-fold increase in cases detected through active surveillance during our study than reported in previous years (105 in 2019 compared to 54 in 2018 and 40 in 2017). *R. sibirica* and *R. raoultii* are the dominant species associated with SFGR in Pavlodar and are also found in local ticks. Improved disease detection and regular testing of humans and ticks in areas with SFGR cases is needed.

**Disclosures:**

**All Authors**: No reported disclosures

